# Construction and validation of a scenario for sedation training in the emergency room for pediatric surgical procedures by in-situ simulation

**DOI:** 10.1590/0100-6991e-20243709-en

**Published:** 2024-07-05

**Authors:** MARCOS MACIEL CANDIDO JUSTINO DOS SANTOS, SARA FITERMAN LIMA, ALEXANDRE SLULLITEL, ROSEMEIRE SIMONE DELLACRODE GIOVANAZZI, FRANCISCO DIEGO NEGRÃO LOPES, MARJORIE DE ARAÚJO VIAN PINHEIRO LIMA, RENÉ SCALET DOS SANTOS, GERSON ALVES PEREIRA

**Affiliations:** 1 - Universidade de São Paulo, Programa de Pós-Graduação em Ciências da Reabilitação HRAC - Bauru - SP - Brasil; 2 - Universidade Federal do Maranhão, Programa de Pós-Graduação em Saúde Coletiva - São Luís - MA - Brasil; 3 - Universidade de São Paulo, Programa de Pós-Graduação ACCEPT - São Paulo - SP - Brasil; 4 - Universidade Federal de Uberlândia, Hospital das Clínicas - Uberlândia - MG - Brasil; 5 - Carefy - Ribeirão Preto - SP - Brasil; 6 - Faculdades Pequeno Príncipe - Curitiba - PR - Brasil

**Keywords:** Simulation Training, Emergency Medicine, Pediatrics, Treinamento por Simulação, Medicina de Emergência, Pediatria

## Abstract

**Introduction:**

sedation and analgesia are fundamental procedures for children undergoing invasive interventions, and complications must be avoided during their implementation. In situ simulation allows, in turn, training in real practice environments to improve the technical and non-technical skills of professionals for such procedures. Although it is a very useful tool, it is often not used due to lack of preparation for its planning and application.

**Objective:**

develop and validate an in situ simulation scenario in pediatric emergency care using sedation to perform an invasive procedure.

**Method:**

descriptive study of construction and content validation of an in situ simulation scenario, using the Delphi method, following the following steps: 1) definition of the problem and selection of experts; 2) development of the initial document; 3) rounds for validation with analysis of responses and feedback (until consensus is reached by the Content Validation Index); 4) final report. Results: The experts indicated suggestions that were duly used and the scenario obtained, in all items, a CVI greater than 80.0%, demonstrating its high validity and reliability. By using experts to validate the scenario, their insights guarantee greater precision and reliability in scenario construction engineering.

**Conclusion:**

It is expected that this study will allow the replication of the scenario in different training contexts, facilitating and encouraging professional training based on a scenario model based on best evidence and practices.

## RESUMO

**Introdução:**

a sedação e analgesia são procedimentos fundamentais para crianças submetidas a intervenções invasivas, devendo-se evitar complicações durante sua realização. A simulação in situ permite, por sua vez, capacitações nos ambientes reais de prática para aprimorar as competências técnicas e não técnicas dos profissionais para tais procedimentos. Embora seja uma ferramenta de grande utilidade, muitas vezes não é aproveitada pelo despreparo para seu planejamento e aplicação.

**Objetivo:**

elaborar e validar um cenário de simulação in situ no atendimento de urgências pediátricas com uso de sedação para realização de procedimento invasivo.

**Método:**

estudo descritivo de construção e validação de conteúdo de um cenário de simulação in situ, por meio do método Delphi, seguindo os seguintes passos: 1) definição do problema e seleção de especialistas; 2) desenvolvimento do documento inicial; 3) rodadas para validação com análise das respostas e feedback (até obtenção do consenso pelo Índice de Validação de Conteúdo); 4) relatório final. Resultados: Os especialistas indicaram sugestões devidamente aproveitadas e o cenário obteve, em todos os itens, um IVC maior que 80,0% demonstrando sua alta validade e confiabilidade. Ao usar especialistas para validar o cenário, seus insights garatem maior precisão e confiabilidade à engenharia de construção dos cenários.

**Conclusão:**

espera-se, que este estudo permita a replicação do cenário em diferentes contextos de formação facilitando e incentivando a capacitação profissional a partir de um modelo de cenário baseado em melhores evidências e práticas.

## INTRODUCTION

In health care, the training of professionals for teamwork, especially for acting in crisis situations and acquiring for knowledge of the multiple aspects involved, results in prevention, mitigation, and learning in the face of risk situations and avoidable adverse events^1^ ^,^ ^2^.

In this sense, sedation and analgesia deserve to be highlighted, since they are important for pediatric patients undergoing invasive procedures, as the need for pediatric sedation has increased considerably, in parallel with the growing volume of procedures performed by different specialists in areas outside the Operating Room (OR), especially in the Emergency Room (ER). Moreover, the administration of sedation has evolved and, in addition to traditional narcotic agents, now includes broader options of agents and routes of administration, increasing the complexity of the challenges faced by professionals during its performance^3^.

Thus, the training of the team to conduct these procedures in children, especially in the ER, is extremely relevant, as the lack of specific training can result in potential risks, including adverse events and complications during the procedure^4^.

In this context, in-situ simulation emerges as an important tool, both to assess the competence and effectiveness of the team, and to provide feedback to improve clinical practice^5^.

In-situ simulation is a training technique that involves the enacting of clinical scenarios, seeking realism by approximating real situations, being carried out in the health care environments themselves, such as ERs, intensive care units, outpatient clinics, ORs, and others. Thus, it allows the team to be trained in an environment like the real one, using the equipment and facilities available in the service itself^6^ ^,^ ^7^.

Its use helps to improve the competence of the multidisciplinary and multiprofessional team, allowing professionals to experience complex and challenging situations in a controlled environment, where it is possible to practice and improve technical and non-technical skills, in addition to training specific procedures. It can therefore help improve the quality and safety of care, especially in stressful situations with time pressure, such as emergencies^5^ ^,^ ^8^. 

Furthermore, with in-situ simulations, it is possible to identify and correct flaws in the care process and in adherence to protocols, reducing errors and complications, in a safe environment for clinical practice. This provides the opportunity to evaluate critical factors, such as communication, leadership, and teamwork, which are essential for this type of care^6^. 

Several studies have highlighted the effectiveness of in-situ simulation to improve staff performance in pediatric emergency situations. One of these was carried out by McLaughlin et al.^9^ and evaluated the impact of in-situ simulation on improving the quality of this care in a children’s hospital. The results indicated that the in-situ simulation significantly improved team effectiveness in several types of competencies, including leadership, communication, and teamwork.

From this perspective, the main objective of this study was to develop and validate an in-situ simulation scenario with the use of sedation for an invasive procedure in a pediatric patient in the emergency room, which can be replicated and used in training of multidisciplinary teams from different health services.

## METHODS

This is a descriptive study of content validation, which worked with an in-situ simulation scenario, whose data are an excerpt from a larger study entitled “The in-situ simulation for evaluation and feedback of pediatric emergency care by a multidisciplinary and multiprofessional team”, object of a dissertation. We used the Delphi method, a research approach to evaluate the content reliability, relevance, and consistency through consensus by a panel of experts. Its steps were: 1) definition of the problem and selection of experts; 2) development of the initial document; 3) rounds for validation with analysis of responses and feedback (until consensus is reached); 4) final report^10^ ^,^ ^11^. 

For the elaboration of the scenarios, we applied the methodology of construction engineering of the simulated cases proposed by Pereira Júnior and Lima^12^, where the planning and organization of the process is carried out in three stages:

I) Choice of the clinical case to be transformed into a simulated activity, emphasizing that at the beginning of structuring a scenario, it is important to define the problem to be worked on, which may be associated with the curricular contents or situations related to the work of professionals in health services, whether these are recognized by them (based on the needs and expectations of those who are preparing the simulated station) or secondary to demands of the health context (administrative, scientific, social, and political studies).
II) Assembly of the 19 items of the order for the simulated station (Table 1), which is the beginning of the transformation of the clinical case into a station, allowing the evaluation of its pertinence, interactions, and feasibility, considering that after the definition of these items, there is already a series of information and elements that allow the visualization of the future station.

**Table 1:** Order Structuring Items for Simulated Scenarios.

1) Theme/content to be addressed (use the content matrix of each area): A title that represents the problem to be developed should be chosen.
2) Target population: Define who the simulation is intended for and consider the students' previous knowledge.
3) Number of participants: define the minimum and maximum.
4) Duration of the scenario: total time planned for all stages, establishing a limit on the duration of the activity with enough time for the participants to achieve the objectives.
5) Learning/assessment objectives: the general objective is what is expected with teaching/assessment. Specific objectives are measures of participant(s) performance, which are usually only available to facilitators/evaluators. The number of specific objectives depends on the complexity and timing of the scenario.
6) General competencies to be developed: knowledge, skills and attitudes expected of the participant at the end of the activity, defining the specific skills to be demonstrated. Competence milestones can be used, which should be mobilised in the development of the station.
7) Type of simulation: define between clinical simulation with the use of simulators (mannequins of various technological complexities), clinical simulation with the use of simulated participants (in general, patients, but they can be family members, members of the professional team, etc.), role play, hybrid simulation, deliberate practice of rapid cycles, in situ simulation, interprofessional simulation, virtual simulation or telesimulation.
8) Clinical case/situation: information on the clinical case to be developed and the tasks to be fulfilled, describing it in a succinct and clear manner, with essential information to achieve the proposed objectives.
9) Lesions/pathologies: define the findings of the anamnesis and physical examination, as well as complementary tests to be explored, associated with specific critical decisions of diagnosis and treatment.
10) Medical procedures to be performed: define the materials and equipment that should be present in the simulated scenario.
11) Distractors: they should be designed with the purpose of aiding in learning and bringing the scenario closer to real conditions, however, they should not excessively divert the participant's attention, distancing him from the proposed objectives.
12) Practice scenario to be simulated: Location within the health care network where the care and/or procedure will be performed (e.g., UBS, outpatient clinic, secondary or tertiary hospital, Emergency Room, ICU or other).
13) Communication problems with patients, family members, and members of the interprofessional team:
The most frequent and relevant situations of conflict that you want to expose the participants to should be used.
14) Ethical and legal conflicts: if they apply to the objectives of the simulation, include the issues that should be discussed, maintaining the realism of the simulated scenario.
15) Interprofessional situation involved: in the use cases, in addition to the specific competencies of each professional category involved in the simulated scenario, common and collaborative competencies must be defined.
16) Estimated level of difficulty: easy, medium or difficult - defined for the moment of training of the participants.
17) Additional information: insert other information that may be useful in the construction of the station to provide greater realism to the simulated scenario.
18) Protocol/consensus guidance for the construction and weighting of the checklist. Preferably they are
based on scientific evidence and widely known to justify the topics and items on the checklist, as well as their score.
19) Expected outcomes: Determine what outcomes are expected for the development of the scenario.

III) Construction of the simulated station (Table 2), using the model for the integral script of the simulated scenario, where there are the instructions of the scenario and tasks of the student/candidate, guidelines to the evaluator/facilitator, list of materials and equipment, map of disposition of furniture and human resources within the physical environment of the simulated station, script of the simulated patient (if the scenic simulation is used), evaluator/facilitator decision flowchart, and standardized evaluation instrument (checklist).

**Table 2:** Structuring items of the complete simulated station.

Definitions prior to the development of the simulated scenario:
• Recording the scenario: define whether the recording will be carried out, as well as the equipment and the person in charge.
• Type of communication between student/candidate and evaluators/facilitators: verbal, written, visual. In the case of selection/sufficiency tests, it is preferable that there is no verbal communication between the evaluator and the candidates.

1) Instructions for the participant/student/candidate: essential information for the clinical case, allowing the participant to understand the care scenario and its function, as well as the definition of the tasks and their duration (establish a duration limit of the activity with enough time for the participants to achieve the proposed objectives). Care should be taken to provide succinct information to be supplemented throughout the development of the simulated scenario. They are divided into two phases:
1.1) Pre-briefing (general guidelines about the simulated activity and 1.2) Briefing (specific guidelines about the simulated scenario).

2) Instructions on the simulated scenario: general description of the complete simulated scenario and definition of items 3, 4 and 5.

3) Checklist for assembling the station: including the physical area that will be used and the arrangement of furniture, materials, equipment and people involved in the scene, for its standardization and reproducibility.
4) Human resources for conducting the scenario: define the different roles to be played in the scenario to establish the number of participants and their prerequisites. In relation to the survey of human resources, facilitators, simulated or standardized patients, operators of technological equipment and others that may be necessary should be included.

5) Material resources: Perform the list of resources, according to the needs and possibilities of the simulated scenario. Example: 1) the physical space to be used (simulation laboratory, health service, or other); (2) simulators (manikins of different technological complexities), if they are used; 3) furniture (bed, stretcher, chair, cabinets, IV support, screen, etc.); 4) equipment (monitor, vacuum cleaner, focus); 6) consumables (gloves, mask, syringes, probes, thermometer, etc.); 7) supporting documentation (referral letters, attendance form, requests for complementary exams, prescriptions, etc.); 8) use of diagnostic and therapeutic resources; and 9) props (clothing, identification documents, medicine packages, previous complementary exams, invasive devices, makeup, blood and secretions, etc.).

6) Guidelines for the simulated participant (patient, family member, team member, etc.): Acting scripts should be prepared and, if necessary, description of the observations for moulage, clothing and props for better characterization and realism.

7) Guidelines and information to the facilitator/examiner/evaluator: sequential and chronological description of the conducts to be taken by the student/candidate.

8) Information about the case and conducts to be taken: Description of the possibilities of conducts and behaviors that the student/candidate can adopt, in order to define his/her action.

9) Flowchart of possible decisions of the stations: to assist the facilitator/evaluator in making decisions about the performance of the participant/student/candidate during the development of the simulated scenario, according to the actions taken, both assertively and in the failures committed.

10) Facilitator /examiner/evaluator checklist: It should contain the appropriate actions/activities to be developed by the participants during the simulated practice, being able to assess technical and/or non-technical skills.

In this study, after the elaboration of the case using the engineering method of the construction of simulated scenarios^12^, we validated the constructed scenario. To this end, we invited 18 professionals, randomly selected based on their expertise in the area, considering articles published in the areas of simulation and pediatric emergency, and research in the curricula available on the Lattes Platform of the National Council for Scientific and Technological Development (CNPq). To avoid bias, we invited professionals from different backgrounds in health.

Twelve experts with training in medicine and nursing and experience in simulation applied to the medical-hospital area in pediatrics and/or emergency and/or intensive care accepted to participate.

The validation process with the committee of experts took place in June 2023, in a virtual environment through the Google Forms^®^ platform, and those who answered the complete instrument were considered as participants.

Each evaluator received the scenario set up with all the items and was instructed to perform the analysis according to the criteria proposed by Pasquali^13^. Thus, they were evaluated by the experts considering 1) feasibility, 2) objectivity, 3) simplicity, 4) clarity, 5) relevance, and 6) accuracy.

We used a Likert-type scale with five response alternatives to evaluate each Pasquali criterion: 1 - Strongly disagree; 2- Disagree; 3- Neither agree nor disagree; 4- Agree; 5- Strongly agree. 

We analyzed the set of answers from these experts to identify the level of agreement between them, and the answers “4” and “5” of each item evaluated were considered for validation purposes, since they indicate agreement by the evaluators. At the end of each item of the simulated scenario, a space was made available for comments and suggestions.

**Figure 1:**
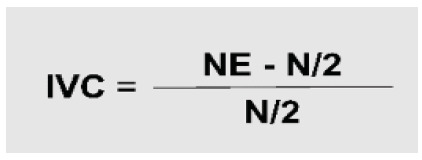
Calculation of CVI based on the concordant answers given by the experts.

The collected data were treated and analyzed using Microsoft Excel^®^ software, version 2019. For the validation of the clinical scenario sections, we calculated the Content Validity Index (CVI), which measures the proportion or percentage of specialists who agree on certain aspects of an instrument^14^ and is computed by summing the responses of the Likert scale and dividing by the total number of responses (Figure 1). Items that obtained 80% or more agreement among experts were considered validated^15^.

In this equation for calculating the CVI, we have the NE, which refers to the number of specialists who agree with a parameter, and the N, which translates the total number of specialists participating in the research^16^.

All ethical aspects were complied with, and the project was approved by the Ethics in Research Committee (CEP) of the Universidade do Oeste Paulista, under opinion No. 5.743.901 and CAAE 63842122.0.0000.5515.

## RESULTS

In the scenario validation stage for the development of the study, the evaluators were asked to fill in sociodemographic information to characterize the participants. We had 12 specialists, of whom eight were women and four men, five from nursing and seven from medicine. All of them had at least six years of professional experience in the area of study and specialization, two with lato senso and 10 with stricto senso^17^. 

The simulated scenario (Table 3) was elaborated according to the methodology of construction engineering of the simulated cases proposed by Pereira Júnior and Lima12 and started with the recognition of the problem or the clinical situation to be addressed. We then chose sedation for the invasive procedure in a pediatric patient in the ER, based on the researchers’ previous care experience.

**Tabela 3:** Cenário simulado de sedação pediátrica.

Simulated clinical case					
1 - Instructions for the participant/student/candidate					
1.1 Orientation for the team - *Pre briefing*					
In the pre-briefing, the facilitator should explain the need for "suspension of disbelief" in order for them to engage in the realism of the clinical case of in situ simulation. They will also check the assembly of the patient and the simulated scenario, so that they are in accordance with what they are used to working on a daily basis. It is also a moment to remove doubts about the behavior during the development of the simulated scenario.					
*With the entire team properly positioned, the facilitator should distribute Form 1 (Professional identification of each team member) and provide the guidelines as follows:*					
*1) An in situ simulation will be carried out to evaluate the multidisciplinary team in the workplace itself, instead of taking everyone to the simulation laboratory.*					
*2) The behavior of the team members should be routine for the clinical situation that will be simulated, and the greater the suspension of disbelief regarding the realism of the scenario was planned, the easier and more natural everyone's performance will be.*					
*3) The simulation will have 5 stages: 1) pre-briefing (which is this current phase), 2) briefing, 3) scenario development, 4) scenario closure and 5) immediate feedback (right after closing) by the local team and, late feedback.*					
*After the above explanations, it should be informed that the pre-briefing is the period in which the facilitator identifies the expectations of the participants, explains how the simulated scenario is set up and what are the roles to be played by the multidisciplinary team during its development, as well as guidance to the participants about the physical space, equipment, consumables and simulator/mannequin.*					
*The facilitator should deliver the material for the assembly of the scenario and the simulated patient, describing it:*					
*The simulated scenario will be set up in a bed in the emergency room with a patient on a stretcher, with a cardiac monitor and pulse oximetry installed, in spontaneous ventilation with a nasal catheter or O2 mask without a reservoir coupled to the flowum, and venous access by peripheral puncture in the left forearm.*					
*The professionals of the team who will provide the care, whose final assembly of the scenario will be at the discretion of the service (nursing, doctors, nursing technician and physiotherapist) will start the scenario to the right of the patient, identified with badges that show the professional function of each*					
*The necessary material should be provided and checked by those involved soon after the start of the simulation scenario. The choice of materials and equipment with their packaging is at the discretion of the team.*					
*For the start of the simulated scenario, the facilitator will be positioned at the lower end of the stretcher. Towards the patient's left foot, the filming assistant will be able to have a full view of the monitor, patient, staff, and devices.*					
1.2 - Staff Orientation - Briefing					
Case report					
The facilitator should explain that the briefing is the moment when the specific clinical case will be presented and the tasks to be performed by the multidisciplinary team will be defined.					
In the briefing, the facilitator should provide information about the clinical case and the task to be performed. Pay special attention to the end goal of the mock case. It is also a time to remove doubts about the clinical case and the execution of tasks.					
It should explain that after the start of the simulated scenario, the performance of the team members should be the same as on a day-to-day basis for the tasks that will be performed.					
At any time, if they have questions about the patient's clinical parameters and/or physical examination data, they should be asked of the facilitator.					
Communication between team members needs to be clear and actions taken aloud so that the facilitator and evaluators can hear.					
If you have any questions, please ask the facilitator.					
The facilitator should inform the closing time of the simulated scenario.					
After the end of the simulated scenario, the facilitator should distribute the updated protocol on sedation/analgesia of children for emergency procedures, providing immediate feedback on the performance of the teams during the execution of the simulated scenario					
If the application of the simulated scenario is recorded, it will be possible to review the video with a new application of the technical and non-technical checklists, comparing the result with the result of the immediate application. Subsequently, it will be possible to provide late feedback, discussing the execution of the tasks by the multidisciplinary team.					
After these explanations, the facilitator should tell the case below:					
*Child, male, 05 years old, 20kg, admitted to the emergency room of a hospital, fasting for 6 hours, requiring chest drainage. It presents with inhalational support of O2, 2L/min, maintaining pulse oximetry around 99%. Minimal respiratory effort, febrile and tachycardic. On physical examination, the patient presented diffuse and bilateral rales and decreased breath sounds at the base of the right hemithorax. Supportive care was performed, the SEPSIS protocol was initiated, and then a simple chest X-ray was performed (attached) showing a 2⁄3 veiling of the right hemithorax (see Imprint 2), compatible with voluminous pleural effusion, with indication of puncture and thoracic drainage. An evaluation of the surgical team was requested to perform the procedure. The care team should provide the necessary preparation and materials for its realization. The surgeon has the function of performing the puncture and thoracic drainage procedure. The sedation/analgesia of the patient will be the responsibility of the pediatrician.*					
Tasks					
In the next 15 to 20 minutes, the multidisciplinary team should perform the following tasks:					
*• Identify and prepare the necessary equipment for the medical procedure.*					
*• Perform patient sedation for the procedure in the emergency room.*					
*• In case of complications, identify and carry out the necessary immediate conducts, including emergency procedures, according to the standard technique.*					
*• Follow the facilitator's instructions.*					

2) Instructions on the simulated scenario					
Description of the scenario					
The simulated scenario will be set up in a bed in the emergency room with a patient on a stretcher, monitored with a cardiac monitor at the head of the bed with pulse oximetry, in spontaneous ventilation with a nasal catheter or O2 mask without reservoir, coupled to a flowmeter and peripheral venipuncture.					
The professionals of the team who will provide care, whose final assembly of the scenario will be at the discretion of the service (nursing, doctors, nursing technician and physiotherapist) will start the scenario to the right of the patient, identified with badges of different colors that show the professional function of each one. After the start of the simulated scenario, the movement of professionals is free.					
The facilitator will be positioned at the lower end of the stretcher, further to the right. Towards the patient's left foot, the filming aid should be positioned, as this will have a complete view of the monitor, patient, team and devices used.					
During sedation for the procedure:					
The crisis situation (distractor) will be initiated by the facilitator and will occur when the stretcher is in an equidistant location between the starting point and the arrival point (radiology sector).					
Final:					
The facilitator will define the closing time of the simulated scenario.					

**3) *Station Assembly Checklist***					
Position of the participants and arrangement of the furniture.					
Initially, the members of the multidisciplinary team should position themselves to the right of the patient (Figure 3). The facilitator will be positioned at the bottom of the stretcher on the right side and the filming assistant will also be at the bottom of the stretcher, but more on the left side so that he can have a privileged view to film all the individuals and processes that will occur.					

4) Human resources to conduct the scenario:					
Available Resources					
*Participants*					
*• Lab coats or private hospital*					
*• Identification of professional function (as per model attached)*					
*• Materials pertinent to the function (stethoscope, goggles, gloves, etc.)*					
*Simulated Patient:*					
*• Children’s shirt*					
*• Children's Bermuda shorts*					
*• Par de chinelo infantil*					

5) Material resources					
*• Fio Mononylon 3-0 and Polypropylene 3-0*					
*• 1 Thorax drain 20, 22 and 24*					
*• 1 Water seal collector*					
*• 1 Suction probe no 06*					
*• 1 O2 nasal catheter*					
*• 3 Scalps of various sizes*					
*• 3 Jelcos nos 20, 22 and 24*					
*• 3 Packets of Gauze*					
*• 1 Pack of Compress*					
*• 4 Tracheal tubes with cuff no. 4.0; 4; 5; 5,0; and 5.5*					
*• 1 Saline solution 500ml*					
*• 1 Medium Micropore Roll*					
*• 1 Roll of Medium Adhesive*					
*• 2 x 5ml syringes*					
*• 2 x 10 ml syringes*					
*• 1 Box of M procedure gloves*					
*• 1 Box of G-procedure gloves*					
*• 1 Patient - low-cost manikin.*					
*• 1 patient bed*					
*• 1 Ambu and mask with reservoir*					
*• 6 vials each identified with the following drugs: Propofol, Fentanyl, Midazolam, Ketamine, Adrenaline and Dobutamine.*					
*• 1 Laryngoscope blade 2*					
*• 1 Laryngoscope slide 3*					
*• 1 Bed Sheet*					
*• 1 Children's shirt*					
*• 1 Children's Bermuda shorts*					
*• 1 Children's Sadalia*					
*• 2 Cell phones or camcorder*					
*• 1 Box of Minor Surgery Supplies*					
Printed					
The following forms are available, as described in the previous items:					
1. Identification of team members					
2. Initial Parameters of Vital Signs Monitor					
3. Simulated patient X-ray					
4. Vital signs monitor parameters during the first complication					
5. Vital signs monitor parameters during the second complication					
6. Vital signs monitor parameters after resolution of the second complication					

6) Guidelines for the simulated participant (patient, family member, team member, etc.)					
6.1) Simulated Patient Information:					
Dummy/Simulator:					
Shop mannequin, already prepared, according to the instructions of the making off.					
*6.1.1 Suit:*					
*• Shirt, shorts and flip-flops.*					
*6.1.2 Devices:*					
*• Nasal catheter or non-rebreathing O2 mask attached to the patient*					
*• Monitor and oximeter attached to the patient*					

6.2) Information about the simulated family member and standardized script					
It could be the father or the mother. You should be extremely concerned about your child's condition, all the time asking for information and what will happen.					
If the team members ask any questions, you should say that you don't know anything because you are separated and the child was with the other spouse.					
You should be instructed by the team members to leave the room during the preparation and procedure, and that you will be informed after the end of the procedure.					
*During the first event:*					
*The simulated family member was close to the door when the first incident began and should try to re-enter the room, in a very nervous way, carrying his cell phone on and talking loudly:*					
*- Why are they taking so long?*					
*- Why doesn't anyone tell me what's going on?*					
*"I need to talk to the staff now, otherwise I'm going to raid the place." I'm filming everything!*					
*Clarifications should be provided to the family member and asked to stop filming and say that there is no authorization for filming in this location. After proper guidance, the simulated family member collects the footage, thanks it and leaves the scene.*					
During the second intercurrence:					
The simulated family member will not interfere, as they will not be present.					

7) Guidance and information for the facilitator/examiner/evaluator:					
Case and Scenario Information					
*- Case Category:*					
Pasive medical procedure in the pediatric emergency room requiring on-site sedation, with complications due to malfunction of venous access, and incomplete infusion of medication with lack of effectiveness in sedation to perform the procedure, presenting agitation. Secondly, with a new venous access, the new infusion of sedative drug will lead to a decrease in the level of consciousness and the need for ventilatory support.					
*- Fulfillment Scenario:*					
Paediatric emergency					
*- Fulfillment Scenario:*					
• Identification of professionals working in the target sector of the simulation.					
• Materials for filming.					
• Simulated family member wanting attention and talking to professionals during the intercurrence.					
• Continuous patient monitoring. The data from the monitor will be narrated and presented by the facilitator.					
• Supplies for peripheral venous access, invasive and non-invasive ventilation, and drugs for sedation.					
• Materials for emergency surgical procedure.					
Purpose of the case and brief description					
Perform a multiprofessional in situ simulation in a daily scenario of performing an invasive procedure in the emergency room, aiming to identify opportunities for improvement and evaluation of the effectiveness of this method for training and feedback of professionals involved in the care of pediatric patients accompanied by family members, specifically in a crisis situation.					
This 5-year-old pediatric patient will undergo an invasive procedure in the pediatric emergency room.					
The initial sedation of the patient will not be effective, there will be psychomotor agitation and impossibility of performing the procedure.					
The simulated family member will enter the scene at this time with cell phone in hand, as he heard the child's complaint. The team should take appropriate action if the family member has not been previously informed.					
In the investigation of the ineffectiveness of sedation, the extravasation of the infused contents in the peripheral venous access should be evidenced, verifying its malfunction.					
After a new venipuncture, the new infusion of sedative drugs will cause a decrease in the level of consciousness and the need for ventilatory support (manual ventilation with AMBU, followed by maintenance with an O2 mask with a reservoir).					
Throughout the service, a family member will show anxiety and request contact with the team.					
Team members should identify what happened and act together to find the most appropriate solutions to the incidents and distractors created at this time.					
All the material necessary for the conduct of the case must have been provided after the start of the simulated scenario.					
Instructions to the facilitator					
1) In the pre-briefing - Guide the team to assemble the simulated patient (low-cost manikin) in an appropriate manner, identifying the role of each team member (Imprint 1).					
2) No briefing - Explicar o caso clínico mostrando o Impresso 2 (Radiografia de tórax) e a tarefa aos membros da equipe multiprofissional. Dar ênfase que o objetivo final é a realização do procedimento de drenagem de tórax, mas que a técnica do procedimento não será motivo de avaliação.					
3) Após o início do cenário simulado:					
a) When professionals ask about the patient's clinical parameters and/or physical examination, Form 3 should be shown with the vital signs - Sat.O2 90%, HR 100 bpm, RR 22 irpm, T 37ºC, BP 110 x 70 mmHg - The facilitator should narrate the parameters.					
b) When those involved in the care are going to administer any medication, they should say out loud the medication and the dose, then the facilitator will answer: "Medication performed".					
4) Define the time to report the two complications:					
a) The facilitator should trigger the crisis situation when the nursing team says that the sedative drug was administered at the request of the pediatrician. The surgeon will be on hand and will arrive as soon as the simulated patient is ready for the procedure.					
b) Verbally explain the first complication, with the clinical picture of psychomotor agitation of the patient and the physical examination of the patient, when requested, and show Form 4 with the vital signs - Sat.O2 90%, HR 120 bpm, RR 28 irpm, T 37ºC, BP 110 x 70 mmHg. To determine the surgeon's inability to proceed with the procedure (Motor agitation, tachycardia (HR of 100 rose to 120 bpm, RR of 22 to 28 ipm), maintaining saturation of 90%) and complaint of pain at the access site.					
c) Talk about extravasation in peripheral access, if asked about it.					
d) Verbally request the dose of medications administered during the procedure.					
e) To state the effectiveness of the procedures performed (new access established and new effective administration of drugs).					
f) Announce the 2nd intercurrence (Imprint 5 - The facilitator narrates and shows the parameters - HR = 120 bpm, RR = 12 irpm and Sat.O2 = 85%, with lowering of the level of consciousness)					
g) *After brief ventilation with a mask and AMBU, the facilitator will confirm the effectiveness of ventilation after its establishment (invasive or not) and should choose to remove the catheter and place a mask with an oxygen reservoir, allowing the procedure to be performed. At this point, you must submit Form 6.*					
h) The facilitator should authorize the sequence of the procedure when the crisis is resolved (patient sedated and with effective ventilation).					
5) Define the closing time of the scenario:					
a) Close the case as soon as the patient is at a good level of sedation, after resolution to the satisfaction of the two distractors, the surgeon will inform the beginning of the procedure.					
b) Close the case, if there is no identification or if there is no solution to the complication after 5 minutes.					
Instructions to evaluators:					
There will be two evaluators per scenario, one responsible for filling out the technical checklist and the other for the non-technical checklist (evaluation of the multiprofessional team).					
The evaluators should be previously trained and should check all the topics and items of each checklist to remove doubts and be familiar with the sequence. These doubts will be cleared up with the facilitator before the start of the simulated scenarios.					
If, during the development of the scenario, there is any doubt about any marking that should be made, a description of the situation encountered and any doubts should be made, and then ask the facilitator at the end of the simulated scenario.					
The initial completion of the checklist can be on paper, precisely so that all doubts are resolved, before entering the data in the computerized form of the checklist or starting with the computerized checklist.					

8) Information about the case and actions to be taken:					
The fundamental items for the procedure should be checked, according to the attached checklist.					
The procedure should be explained to the simulated family member at the beginning of the scenario, and asked to leave the room momentarily.					
Let the team make your identification (Printout 1), organize themselves as usual and make the initial infusion of the drugs used and prepare for the surgeon to perform the procedure. They should be familiar with the clinical case and the chest X-ray (Printout 2) that shows the need for pleural drainage.					
When the professionals ask about the clinical parameters and/or physical examination of the patient, Form 4 - Sat.O2 90%, HR 120 bpm, RR 28 irpm, T 37ºC, BP 110 x 70 mmHg should be delivered. Thus, the presence of psychomotor agitation, tachycardia (HR of 100 rose to 120 bpm, RR of 22 to 28 bpm), maintaining saturation of 99%) and complaint of pain at the access site should be noted.					
- When those involved in the care are going to administer any medication, they should say out loud the medication and the dose, then the facilitator will answer:					
- Medication performed.					
- The possibilities for ineffective sedation, including venous device dysfunction, should be checked.					
- The simulated family member who will act as another distractor of the communication of accomplishment entering the scenario with the cell phone on, must instigate the following aspects.					
- Why are they taking so long?					
- Why doesn't anyone tell me what's going on?					
"I need to talk to the staff now, otherwise I'm going to raid the place." Look, I'm filming everything.					
Clarifications should be provided to the family member and asked to stop filming and say that there is no authorization for filming in this location.					
- After proper guidance, the simulated family member collects the footage, thanks it and leaves the scene.					
- Another access should be arranged and the medication should be taken again.					
The Facilitator should confirm aloud the drug infusion and expose the new clinical picture and show the vital signs (Imprint 5 - The facilitator shows and narrates the parameters - HR = 120 bpm, RR = 12 ipm and Sat.O2 = 85%, with lowering of the level of consciousness)					
- After brief ventilation with a mask and AMBU, the facilitator will confirm the effectiveness of ventilation after its establishment (invasive or not) and should choose to remove the catheter and place a mask with an oxygen reservoir, allowing the procedure to be performed (Imprint 6).					
The facilitator should inform that the case is closed after the surgeon indicates the start of the procedure.					
The facilitator must inform that the case is closed if it is not resolved within 05 minutes after the second intercurrence.					

9) Flowchart of possible decisions of the stations It is a graphic representation of the scenario, containing the possible decisions of the participants, and guiding the facilitator/evaluator, in the sequence of conducting the case, based on these decisions					

10) Technical & Non-Technical Checklist (Topics & Items)					
**10.1) *Technical Checklist***					
The Evaluators should be selected together with the facilitator and will be present from the beginning of the instructions to the multidisciplinary team. It is important to instruct the evaluators about the need to note the start times of each topic and item marked in the right side column of the checklist, with time zero (T0) being the moment when the facilitator announces the beginning of the simulated scenario. The other times (T1 to T15) should be counted and noted from T0 onwards.					

Evaluation indicators					T0=
A	Check Items for the Procedure	Yes		No	T1=
1	Separation of the materials to be used to perform the invasive procedure.				
2	Separation of materials to be used in complications involving sedation in pediatrics.				
3	Adequate and private space was provided for the procedure.				
4	The multidisciplinary team was available at the time of the procedure.				
B	Orientations for family members	Yes		No	T2=
1	He reassures family members about the procedure and asks them to wait outside the room until the end of the procedure.				
2	Approach to the stressed family member when the 1st complication occurs. *He provided an explanation of the situation and the need to wait outside the emergency room while the procedure is performed.*				
3	It advises the family member that after the procedure they will talk about the procedure, prognosis and complications.				
C	Use of sedation/analgesia scales	Yes		No	T3=
1	Michigan				
2	Ramsay				
3	Ramsay modificada				
4	Confort				
5	Other:				
D	Parameters for sedation * Inappropriate administration of the drug is outside the established dosages	Did not	Inadequate	Adequate	T4=
1	Propofol (P) 1 a 3 mg/kg				
2	Fentanil (F) 20 a 30 mcg (0,02 a 0,03mg ou 0,4 a 0,6mL), EV, a cada 10 a 12kg de peso corporal.				
3	Midazolam(M) 0,1 a 0,4 mg/kg				
4	Cetamina (C) 0,5 a 2mg (EV/IO) 2-4mg/kg (IM)				
5	Etomidato (E) 0,2 a 0,4 mg/kg				
E	Technical Skills	Did not	Inadequate	Adequate	
1	Infusion of medication into venous access				T5=
2	Valuation of psychomotor agitation and alterations of vital parameters				T6=
3	Identification of the first complication with the patient				
4	Identification of venous access dysfunction				
5	Obtaining new peripheral vascular access				T7=
6	Obtenção de novo acesso vascular periférico				T8=
7	Performed the infusion of new sedative/analgesic drugs *Propofol (P) 1 a 3 mg/kg* *Fentanil (F) 20 a 30 mcg (0,02 a 0,03mg ou 0,4 a 0,6mL), EV, a cada 10 a 12kg de peso corporal.* *Midazolam(M) 0,1 a 0,4 mg/kg* *Cetamina (C) 0,5 a 2mg (EV/IO) 2-4mg/kg (IM)* *Etomidato (E) 0,2 a 0,4 mg/kg*				T9=
8	Identification of the second complication with the patient (decreased consciousness, hypoxia and hypoventilation)				T10=
9	Identification of the need for ventilation				T11=
10	Momentary ventilation with AMBU and O2 mask with reservoir				T12=
11	Replacement of the O2 catheter for a mask with a reservoir at 5 L/min				T13=
12	Identification of normalization of vital signs				
13	Identification of the appropriate level of sedation and analgesia				T14=
14	Resumption of the possibility of performing the surgical procedure				T15=
					
**10.2) *Non-technical* checklist**					
MHPTS Scale* Part I		Evaluation of the team's performance in each item during the actions					
Evaluation indicators		0 Never or rarely	1 Inconsistent		2 Consistent
Consistently mark when the many qualities described in each item have been demonstrated in the actions					
1	A leader is clearly recognized by all team members.				
2	The team leader ensures that an appropriate balance is maintained between command authority and team member participation.				
3	Each team member demonstrates a clear understanding of their assignments.				
4	The team guides each one to meet all significant clinical indicators during procedures/interventions.				
5	When team members are actively engaged with the patient, they verbalize their activities out loud.				
6	Team members repeat or paraphrase instructions or clarifications to indicate that they heard correctly.				
7	Team members indicate established protocols and checklists for the procedure/intervention.				
8	All team members are properly involved and participate in the activity.				
Items 9-16 may be marked “NA (not applicable)” if there are no situations in which these types of responses are necessary.					
MHPTS Scale* Part II		Evaluation of the team's performance in each item during the actions					
Evaluation indicators		0 Never or rarely	1 Inconsistent	2 Consistent	NA Not applicablel
The items below can be marked as “NA (not applicable)”, if necessary, according to the situations demonstrated or not by the teams					
9	Disagreements or conflicts between team members are addressed without loss of control of the situation.				
10	When appropriate, roles are swapped to address urgent issues or emerging events.				
11	When instructions are unclear, team members acknowledge their lack of understanding and ask for repetition and clarification.				
12	Team members acknowledge - in a positive way - guidance aimed at avoiding or containing errors, or seeking clarification.				
13	Team members pay attention to actions that they feel could cause errors or complications.				
14	Team members account for potential errors or complications with procedures by avoiding them.				
15	When statements intended to prevent or contain errors or complications do not elicit a response to avoid or contain the error, team members persist in finding an answer.				
16	Team members ask each other to help each other, before or during periods of task overload.				

*Mayo High Performance Teamwork Scale validated for Brazil

**Figure 2:**
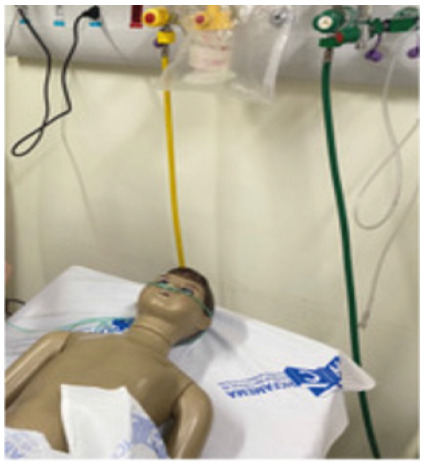
Shop mannequin adapted to the scenario.

The scenario was proposed using a mannequin of low complexity, technology, and cost, with the assembly of a bed in the emergency room with a patient on a stretcher, with a cardiac monitor, pulse oximetry, in spontaneous ventilation, with a nasal catheter or O2 mask without a reservoir coupled to a flowmeter, and peripheral venipuncture. In this proposal, if the service does not have a simulated children’s mannequin, one can opt for a low-cost mannequin (shop children’s mannequin, prepared according to the instructions of the making off - Figure 2, available at the link https://drive.google.com/file/d/1FMjE5iDVxxQeSyAIAtMXu9hGYvsvBy4U/view?usp=sharing ).

We should note that the setting of the scenario must ensure the necessary realism for the proposed simulation, and the mannequin/simulator must wear a shirt, shorts, and slippers, with a nasal catheter or non-rebreathing O2 mask, and the multiparametric monitor and pulse oximeter attached to the patient.

Team professionals should be at the discretion of the service, and may include nurses, physicians, physiotherapists, nursing technicians, and others. 

Before starting the simulated scenario, the pre-briefing should be carried out, at which time the facilitator can identify the expectations of the participants and guide them about the physical space, equipment, materials, and simulators, as well as the behavior they may have in the development of the simulated scenario.

**Figure 3:**
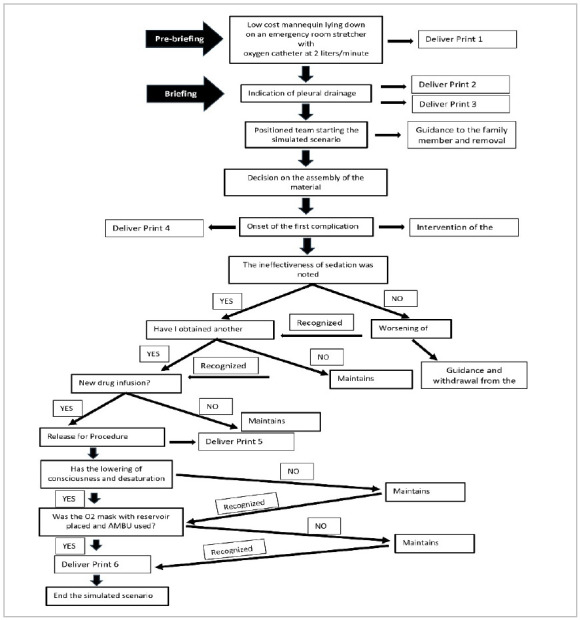
Flowchart for evaluator/facilitator decision-making.

In the pre-briefing, with the entire team properly positioned, the facilitator should offer the following guidelines:

a) An in-situ simulation will be carried out to evaluate the multidisciplinary team in the workplace, instead of taking everyone to the simulation laboratory.
b) The behavior of the team members should be routine for the clinical situation that will be simulated, and the greater the “suspension of disbelief” regarding the realism of the planned scenario, the easier and more natural everyone’s performance will be^18^.
c) Explanation of the simulated scenario set up and of the roles to be played by the multidisciplinary team, as well as guidance on the physical space, equipment, consumables, and simulator/mannequin.
d) The simulation will have five stages: I) pre-briefing (the current phase), II) briefing, III) scenario development, IV) scenario closure, and V) immediate feedback (right after closing). If the simulated scenario is being recorded, the feedback may also be delayed.
e) The development of the scenario should follow the decision flowchart of the evaluator/facilitator (Figure 3) and the standardized evaluation checklist (Chart 3).

The facilitator must deliver the material for the assembly of the scenario and the simulated patient, describing it as follows: the simulated scenario is mounted on a bed in the emergency room with the patient on a stretcher, with a cardiac monitor and pulse oximetry installed, on spontaneous ventilation with a nasal catheter or O2 mask without a reservoir coupled to the flowmeter, and venous access by peripheral puncture on the left forearm. 

The team professionals who will provide the care, whose organization will be at the discretion of the service (nursing, doctors, nursing technicians, and physiotherapists), must be identified by badges of different colors.

The necessary material should be provided and checked by those involved soon after the start of the simulation scenario. The choice of materials and equipment with their packaging is at the discretion of the team.

**Figure 4:**
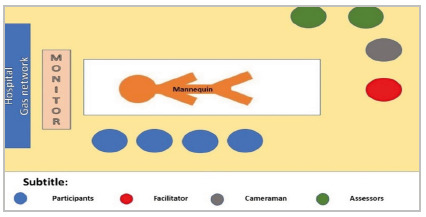
Orientation to the team’s position in the scenario.

For the beginning of the simulated scenario, the professionals participating in the simulation scenario will be to the right of the patient, identified with badges that show the professional function of each. The facilitator will be positioned at the lower end of the stretcher. Towards the patient’s left foot, the filming assistant will be able to have a full view of the monitor, patient, staff, and devices. The two evaluators, one with a technical checklist and the other with a non-technical one, are on the opposite side of the participants (Figure 4).

[Fig f5]

**Figure 5 f5:**
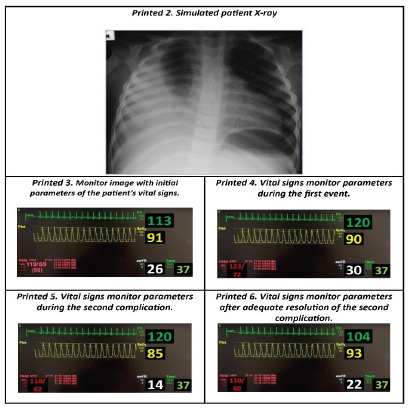
Printouts to be used in the application of the simulated scenario.

In the case of a sedation scenario for an invasive procedure in a pediatric patient, the parameters of the monitor become especially important to work with fidelity and realism, and can be simulated through a high-tech mannequin system, by an application projected on a television or monitor, delivered in printouts, or even narrated by the facilitator. In this scenario, we decided to include the printed format to facilitate its reproduction.

In the briefing, when the specific orientations of the scenario that would be developed for the multidisciplinary team should be carried out, the clinical case, the tasks to be performed, and the duration of the simulated scenario should be briefly presented, with the reading of the following case:

*Child, male, five years old, 20kg, admitted to the emergency room of a hospital, fasting for six hours, requiring chest drainage. He presents with inhalational support of O2 at 2L/min, maintaining pulse oximetry around 99%. Minimal respiratory effort, febrile and tachycardic. On physical examination, the patient presented diffuse and bilateral rales and decreased breath sounds at the base of the right hemithorax. Supportive care was performed, the sepsis protocol was initiated, and then a simple chest X-ray was performed (Printout 2), which showed a 2⁄3 veiling of the right hemithorax, compatible with voluminous pleural effusion, with indication for puncture and thoracic drainage. An evaluation of the surgical team was requested to perform the procedure. The care team should provide the necessary preparation and materials for its execution. The surgeon has the function of performing the puncture and thoracic drainage procedure. The sedation/analgesia of the patient will be the responsibility of the pediatrician.*

The definition of the task or tasks is an essential step to achieve execution success in the scenario and should be set based on the learning or assessment objectives. They should have clear language, be direct, and state what should be done and in how much time. 

Thus, the activities proposed for this scenario were:

In the next 15 to 20 minutes, the multidisciplinary team should perform the following tasks:

- Identify and prepare the necessary equipment for the medical procedure.
- Perform patient sedation for the procedure in the emergency room.
- In case of complications, identify and perform the necessary immediate conducts, including emergency procedures, according to the standard technique.
- Follow the facilitator’s instructions.

The facilitator should inform that the monitoring data will be presented in the form of printed materials.

After the start of the simulated scenario, the facilitator should pay attention to:

a) When the professionals ask about the clinical parameters and/or physical examination of the patient, Printout 3 should be shown with the vital signs - O2 sat. 91%, HR 113 bpm, RR 26 irpm, T 37ºC, BP 119 x 69 mmHg; the parameters should be narrated;
b) When those involved in the care are going to administer any medication, they should say out loud the medication and the dose, then the facilitator will answer: “Medication performed”;
c) The moment to inform the expected complications.

Team members are expected to explain the procedure to the simulated family member at the beginning of the scenario, and ask them to leave the room momentarily, in which case space should be guaranteed for the team to organize themselves in case they perform this movement.

The facilitator must be aware of the critical actions that must be carried out by the participants, since they signal whether the objectives of the simulated scenario are being achieved. 

The first expected complication is the malfunction of the venous access, with extravasation of the infused contents and incomplete infusion of the medication, with lack of effectiveness in sedation for the execution of the procedure, resulting in psychomotor agitation, with impossibility of performing the procedure. The facilitator should trigger the crisis when the nursing team verifies that the sedative drug was administered at the request of the pediatrician. The surgeon will be on hand and will arrive as soon as the simulated patient is ready for the procedure.

The facilitator should verbally explain the first complication, with the clinical picture of psychomotor agitation of the patient, and complaint of pain at the site of access and the physical examination of the patient, when requested, and show the Printout 34 with the vital signs - O2 sat. 90%, HR 120 bpm, RR 30 irpm, T 37ºC, PA 123 x 72mmHg. 

The team should determine the surgeon’s inability to continue the procedure - motor agitation, tachycardia (HR rises from 100 to 120 bpm, RR, from 22 to 28 ipm), maintaining saturation of 90% - and complaint of pain at the site of access. 

The facilitator should talk about extravasation in the peripheral access if questioned about it and verbally request the dose of the medications administered during the procedure. With the expected actions of the care team (new access established and new effective administration of drugs), the facilitator should report on the effectiveness of the procedures performed.

Throughout the service, the simulated family member, guided by the script:

*Will demonstrate anxiety and request contact with the team. At the moment of the first complication, he will enter the scene with a cell phone in his hand, because he heard the patient’s complaint, and will start to instigate the following aspects: a) “Why are they taking so long?” b) “Why doesn’t anyone tell me what’s going on?” and c) “I need to talk to the team now, otherwise I’m going to invade the place”. The team should take appropriate action if the family member has not been previously informed. If the team has not taken any action, the simulated family member must enter the set with his cell phone on and say, “Look, I’m filming everything.” The team should provide clarification to the family member, ask him to stop filming, and say that there is no authorization for filming in this location. After proper guidance, the family member puts the cell phone away, thanks, and leaves the scene.*

The second complication, after obtaining a new venous access, is the lowering of the level of consciousness and the need for ventilatory support (manual ventilation with AMBU, followed by maintenance with an O2 mask with a reservoir), with the infusion of a new sedative drug.

When announcing the second complication, the facilitator hands out Printout 5 and narrates the following parameters: HR 120 bpm, RR 14 irpm, O2 sat. 85%, T 37ºC, BP 100 x 62mmHg, with lowered level of consciousness.

The expected action of the team is a brief period of ventilation with a mask and AMBU, changing the oxygen delivery device, removing the catheter, and placing the mask with a reservoir at 5 L/minute. In this case, the facilitator will confirm the effectiveness of ventilation by handing out Printout 5, which shows the improvement in vital signs: PO2 93%, HR 104 bpm, RR 22 irpm, Tax 37ºC, BP 110x60mmHg. 

When the second complication is resolved (patient sedated and with effective ventilation), the facilitator should authorize the procedure, ending the scenario.

All the necessary materials during the cases’ care must be provided for the development of the simulated scenario. 

The resources available for the simulated in-situ scenario are:

- Materials for emergency surgical procedures;
- Simulated family member wanting attention and talking to professionals during the complication;
- Material for continuous monitoring of the patient (the monitor’s data will be narrated and presented in printouts by the facilitator);
- Identification of material for professionals;
- Filming materials;
- Professionals working in the target sector of the simulation;
- Supplies for peripheral venous access, invasive and non-invasive ventilation, and sedation.

The goal is to perform the chest drainage procedure in the emergency room, but the procedure technique will not be evaluated.

Regarding the closure of the scenario, the facilitator should inform that the simulated case will be closed as soon as the patient’s vital signs are close to normal and at a good level of sedation, after the satisfactory resolution of the two complications, at which time the facilitator should verbalize that the surgeon can start the procedure. The case should also be closed if there is no identification or if there is no solution to the second complication after five minutes.

The experts’ individual evaluation of the scenario is shown in Table 4, where all items had a CVI equal to or greater than 0.8, which indicates the validation of the data by the experts. The agreement among the experts ranged from 91 to 100%, pointing to contents’ reliability, relevance, and consistency^16^. 

**Tabela 4:** Experts’ scores for the criteria of each item, with the respective content verification indexes and agreement percentage.

Criteria	Notes from Specialists												IVC¹	%²	
	01	02	03	04	05	06	07	08	09	10	11	12		
Item 01 (Guidelines for setting up the scenario)														
Clarity	5	5	5	5	5	5	5	5	5	5	5	4	1,0	91
Pertinence	5	5	5	5	5	5	5	5	5	5	5	4	1,0	91
Simplicity	5	5	5	5	5	5	5	5	5	5	5	2	0,8	91
Precision	5	5	5	5	5	5	5	5	5	5	5	4	1,0	91
Practicability	4	5	5	5	5	5	5	5	5	5	4	4	1,0	100
Relevance	5	5	5	5	5	5	5	5	5	5	5	5	1,0	100
Item 02 (Guidelines for the team - pre-briefing)														
Clarity	5	5	4	5	5	5	5	5	5	5	5	4	1,0	100
Pertinence	5	5	5	5	5	5	5	5	5	5	5	4	1,0	91
Simplicity	5	5	4	5	5	4	4	5	5	5	5	2	0,8	91
Precision	5	5	4	5	5	4	4	5	5	5	5	4	1,0	100
Practicability	5	5	5	5	5	5	5	5	5	5	4	3	0,8	91
Relevance	5	5	5	5	5	5	4	5	5	5	5	5	1,0	100
Item 03 (Guidelines for the team - briefing)														
Clarity	5	5	5	5	5	5	5	5	5	5	5	4	1,0	100
Pertinence	5	5	5	5	5	5	5	5	5	5	5	5	1,0	100
Simplicity	5	5	5	5	5	5	4	5	5	5	5	4	1,0	100
Precision	5	5	5	5	5	4	4	5	5	5	5	4	1,0	100
Practicability	5	5	5	5	5	5	5	5	5	5	4	4	1,0	100
Relevance	5	5	5	5	5	5	5	5	5	5	5	5	1,0	100
Item 04 (Guidelines for the facilitator)														
Clarity	4	5	5	5	5	5	5	5	5	5	5	4	1,0	100
Pertinence	4	5	5	5	5	5	5	5	5	5	5	4	1,0	100
Simplicity	5	5	5	5	5	5	5	5	5	5	5	4	1,0	100
Precision	5	5	5	5	5	4	5	5	5	5	5	4	1,0	100
Practicability	4	5	5	5	5	5	4	5	5	5	4	4	1,0	100
Relevance	5	5	5	5	5	5	5	5	5	5	5	5	1,0	100
Item 05 (Printed)														
Clarity	5	5	4	5	5	5	5	5	5	5	5	4	1,0	100
Pertinence	5	5	5	5	5	5	5	5	5	5	5	5	1,0	100
Simplicity	5	5	5	5	5	5	4	5	5	5	5	4	1,0	100
Precision	5	5	4	5	5	5	3	5	5	5	5	5	0,8	100
Criteria	Notes from Specialists												IVC¹	%²
	01	02	03	04	05	06	07	08	09	10	11	12		
Practicability	5	5	5	5	5	5	5	5	5	5	5	4	1,0	100
Relevance	5	5	5	5	5	5	5	5	5	5	5	5	1,0	100
Item 06 (Information to Simulated Patients)														
Clarity	5	5	5	5	4	5	5	5	5	5	5	5	1,0	100
Pertinence	5	5	5	5	5	5	5	5	5	5	5	5	1,0	100
Simplicity	5	5	5	5	5	4	5	5	5	5	5	4	1,0	100
Precision	5	5	5	5	4	4	5	5	5	5	5	4	1,0	100
Practicability	5	5	5	5	5	5	5	5	5	5	5	4	1,0	100
Relevance	5	5	5	5	5	5	5	5	5	5	5	4	1,0	100
Item 07 (Information to evaluators)														
Clarity	5	5	5	5	5	5	5	5	5	5	5	4	1,0	100
Pertinence	5	5	5	5	5	4	5	5	5	5	5	4	1,0	100
Simplicity	5	5	5	5	5	5	5	5	5	5	5	4	1,0	100
Precision	5	5	5	5	5	4	5	5	5	5	5	4	1,0	100
Practicability	5	5	5	5	5	4	5	5	5	5	5	4	1,0	100
Relevance	5	5	5	5	5	4	5	5	5	5	5	5	1,0	100
Item 08 (Technical Checklist Topics)														
Clarity	4	5	5	5	5	5	5	5	5	5	5	4	1,0	100
Pertinence	5	5	5	5	5	4	5	5	5	5	5	3	0,8	91
Simplicity	4	5	5	5	5	4	5	5	5	5	5	2	0,8	91
Precision	5	5	5	5	5	5	5	5	5	5	5	3	0,8	91
Practicability	4	5	5	5	5	5	5	5	5	5	5	3	0,8	91
Relevance	5	5	5	5	5	5	5	5	5	5	5	5	1,0	100
Item 09 (Technical Checklist - Items)														
Clarity	5	5	5	5	5	4	5	5	5	5	5	4	1,0	100
Pertinence	5	5	5	5	5	5	5	5	5	5	5	5	1,0	100
Simplicity	5	5	5	5	5	4	4	5	5	5	5	4	1,0	100
Precision	5	5	5	5	5	5	4	5	5	5	5	4	1,0	100
Practicability	4	5	5	5	5	4	5	5	5	5	5	4	1,0	100
Relevance	5	5	5	5	5	5	5	5	5	5	5	5	1,0	100

1- IVC: Content Verification Index; 2 - %: Percentage of agreement

The experts also indicated eight suggestions for improvements for the clinical case and five for the checklist, all of which were duly incorporated.

## DISCUSSION

Clinical simulation is an active teaching-learning strategy that reproduces real-world situations and helps learners to consolidate knowledge and develop technical and non-technical skills^19^. It offers a practical experience, recreating relatively common situations (airway obstruction, laryngospasm, and bronchospasm) and rare situations (cardiovascular collapse, aspiration, and anaphylaxis), ranging from the simple recreation of clinical scenarios^20^ ^-^ ^22^.

Simulation can develop the skills of the professionals who will use it, support the identification of means to predict and prevent adverse events, and better develop teamwork, since the ability to use, organize, and direct a team is important in crisis management^22^.

In-situ simulation has been increasingly used in different medical specialties and training contexts, improving teamwork and individual learning, and offering greater realism and transferability at a lower cost, as it does not require expenses with the implementation and maintenance of a simulation center. In addition, its use improves performance in real clinical scenarios, helping to reveal important latent risks and allowing the implementation of corrective measures^23^.

However, it has often been applied without a specific design that considers the educational needs, clinical demands, and available resources, which minimizes its impacts^24^.

A recent review study on the use of in-situ simulation worldwide concluded that there is still much to expand as to the use of this resource, especially in Brazil, which reinforces the relevance of the scenario presented^23^.

Studies have shown that when simulation is well planned and meaningful to the participants, it increases the level of confidence and self-efficacy, reinforces knowledge, improves skills for care, communication, and interpersonal relationships, develops critical thinking and clinical judgment, promotes empathy, and allows reflection on actions^24^ ^,^ ^25^. 

It is noteworthy that the structuring of the simulated scenarios requires prior, intentional, systematic, and thorough planning of the proposed activity^12^ ^,^ ^17^, and its validation is of great relevance, as it guarantees the content’s quality and validity, in addition to supporting the objectives and expected results^26^ ^,^ ^27^.

The overall goal of scenario engineering should be to facilitate the delivery and achievement of a set of clear learning outcomes while maintaining fidelity at the highest possible level^4^.

At the beginning of structuring a scenario, it is of great importance to define the problem to be addressed and the simulation’s target audience, and in this sense, sedation and analgesia are important steps for pediatric patients undergoing invasive procedures^28^. The objective of sedation and analgesia is to achieve a state of consciousness that allows the patient to remain with the airway open and minimize pain and discomfort^28^ ^,^ ^29^.

Pediatric sedation continues to be a growing challenge, as the need for the procedure to be performed by different medical specialties in different hospital sectors increases concomitantly with the emergence of new agents and access routes^3^ ^,^ ^30^. 

In emergency medicine, this is a common practice supported by the fact that physicians already have skills in sedation, airway management, and cardiovascular resuscitation^3^ ^,^ ^31^. 

Training of the team to conduct sedation for invasive procedures in children, especially in the emergency room, is of great importance, because the lack of specific training can result in potential risks, including adverse events and complications during the procedure^28^. 

Among the potential complications of pediatric sedation are: respiratory depression, which is the most common and can cause hypoxemia, apnea, and even cardia arrest; cardiovascular instability, with changes in heart rate, blood pressure, and cardiac output (particularly dangerous in children with previous cardiovascular disease); allergic reactions to some sedatives; increased risk of aspiration, especially in children with gastrointestinal or respiratory problems; delayed recovery in response to some agents; and rare neurological complications, including seizures or stroke. Moreover, medication errors during the administration of sedation can lead to incorrect dosage, discomfort, anxiety, and suffering due to inadequate sedation, and excessive sedation, with consequent respiratory depression and cardiovascular instability^32^ ^-^ ^34^.

Several studies therefore highlight the need for training emergency physicians to perform pediatric sedation, ensure the safety and efficacy of the procedure, and reduce the risk of adverse events^3^ ^,^ ^35^ ^,^ ^36^. 

Thus, to ensure the safe and efficient administration of sedation in a manner adapted to the individual needs of each child, the team must acquire essential skills and knowledge to promptly identify and address any eventuality, which can be properly worked on in the simulation^28^.

The construction and validation of the in-situ simulation scenario on emergencies common to pediatric care practice may support future training and evaluations aimed at the multidisciplinary team involved in this theme. However, we point out as limitations of the study the non-validation of the results, which is optional for the method used, and consequently the non-presentation of the data resulting from the application of the scenario. Nonetheless, we emphasize that the application of the scenario will consist of a future stage of the study^37^ ^,^ ^38^. 

## CONCLUSION

In this work, we constructed an in-situ simulation scenario and validated it in situations of pediatric emergency care with sedation for surgical procedures, considering the relevance of the team’s preparation to conduct these procedures.

Twelve specialists, with extensive experience in the areas of clinical simulation, participated in the validation process and the adapted simulation scenario proved to be adequate, obtaining an overall CVI value >0.80 among the specialists, pointing to the reliability of the scenario. 

There was a consensus on the consistency of the proposed scenario, and its replication by other professionals, facilitators, teachers, and scholars, will impact on time savings in planning and ensure greater reliability in the training process.

We hope that this study will allow the use of the scenario in different training contexts, facilitating and encouraging professional training based on a scenario model grounded on best evidence and practices.
